# Effects of pseudoephedrine on parameters affecting exercise performance: a meta-analysis

**DOI:** 10.1186/s40798-018-0159-7

**Published:** 2018-10-05

**Authors:** Maria D Gheorghiev, Farzad Hosseini, Jason Moran, Chris E Cooper

**Affiliations:** 1 Centre for Sports and Exercise Science, School of Biological Sciences, University of Essex, Colchester, UK; 2 School of Sport, Rehabilitation and Exercise Sciences, University of Essex, Colchester, UK; 3 Department of Sport, Hartpury University, Gloucestershire, UK; 4 Faculty of Medicine, Carol Davila University of Medicine and Pharmacy, Bucharest, Romania

**Keywords:** Performance-enhancing drugs, Anti-doping, Training, Sport, Pseudoephedrine, Stimulant

## Abstract

**Background:**

Pseudoephedrine (PSE), a sympathomimetic drug, commonly used in nasal decongestants, is currently banned in sports by the World Anti-Doping Agency (WADA), as its stimulant activity is claimed to enhance performance. This meta-analysis described the effects of PSE on factors relating to sport performance.

**Methods:**

All included studies were randomised placebo-controlled trials and were conducted in a double blind crossover fashion. All participants (males and females) were deemed to be healthy. For the primary analysis, standardised mean difference effect sizes (ES) were calculated for heart rate (HR), time trial (TT) performance, rating of perceived exertion, blood glucose, and blood lactate.

**Results:**

Across all parameters, effects were trivial with the exception of HR, which showed a small positive increase in favour of PSE ingestion (ES = 0.43; 95% confidence interval: − 0.01 to 0.88). However, subgroup analyses revealed important trends. Effect sizes for HR (increase) and TT (quicker) were larger in well-trained (VO_2_ max (maximal oxygen consumption) ≥ 65 ml/kg/min) and younger (< 28 years) participants, for shorter (< 25 mins) bouts of exercise and when PSE was administered less than 90 min prior to performance. There was evidence of a dose-response effect for TT and HR with larger doses (> 170 mg) resulting in small (ES = − 0.24) and moderate (ES = 0.85) effect sizes respectively for these variables.

**Conclusions:**

We conclude, however, that the performance benefit of pseudoephedrine is marginal and likely to be less than that obtained from permitted stimulants such as caffeine.

## Key points

- Pseudoephedrine use exerts an effect on heart rate, but there is no effect on time trial performance, perceived effort, or biochemical markers (blood glucose and blood lactate).
- Effects could be more apparent in just those athletes of most concern to anti-doping agencies: younger and well-trained athletes.
- Any performance benefit of pseudoephedrine is marginal and certainly less than that obtained through permitted stimulants such as caffeine

## Background

Pseudoephedrine (PSE) is a sympathomimetic amine derived from the plant genus *Ephedra*, most commonly used at therapeutic levels (60 mg) to relieve nasal congestion. The principal mechanism of action of PSE relies on the indirect stimulation of peripheral α_1_-adrenergic receptors, although it also has some ability to stimulate cardiac β receptors. This causes vasoconstriction at the level of the nasal mucosa, therefore reducing the blood flow to the nasal cavity and decreasing inflammation [1]. Despite being the optimal drug for this condition in many countries, access to PSE is restricted as it is a precursor material for the illegal manufacture of amphetamine. Instead, the less effective phenylephrine is favoured [2].

Due to its similarity in structure with ephedrine and other central nervous system stimulants, there has been speculation that PSE may also exert ergogenic effects. Previously reviewed by Trinh et al. [3], these effects include increased systolic and diastolic blood pressure and heart rate, vasoconstriction in the cutaneous vessels, vasodilation in the skeletal muscle, and breakdown of glycogen in the liver and muscle. Increased glycogenolysis could lead to increased glucose supply when it is limiting in exercise, whereas the proposed inotropic and chronotropic effects on the heart could raise cardiac output, promoting blood flow to working muscle and potentially improving performance [4, 5]. Regardless of the theoretical advantages of PSE, the results of studies to document its efficacy as an ergogenic agent are equivocal. Many studies have found little, or no, ergogenic effect [6–12], although some researchers reported larger-than-therapeutic doses to be effective in enhancing performance [13–15].

This inconsistency in research findings is reflected in the variability in PSE’s regulation by anti-doping agencies and other sporting bodies. The International Olympic Committee banned PSE until 2004 when the World Anti-Doping Agency (WADA) removed it from the prohibited list. In 2010, after a monitoring program that suggested increased use by athletes following the lifting of the ban, it was banned again. However, pseudoephedrine is not banned in professional sports that do not follow the WADA prohibited list, one example being ice hockey, in which it is used extensively. Even one pill a day used as a decongestant can trigger a positive drug test, and this can lead to unfortunate consequences such as the banning of Swedish National Hockey League player Nicklas Backstrom from the gold medal game of the Sochi 2014 Winter Olympics.

A recent systematic review by Trinh et al. [3] used qualitative analysis to suggest that only higher doses of PSE are likely to enhance performance. The authors argued the limited studies available were too heterogeneous to perform a quantitative meta-analysis to supplement their findings. We performed a new review, finding seven additional articles not included by Trinh et al. [3]. The inclusion of these articles made a quantitative analysis possible, not just in terms of PSE’s potential effect on performance, but also on physiological (heart rate [HR]), biochemical (blood glucose [GLU], blood lactate [LAC]), and psychological (RPE) markers that could inform on the proposed mechanism of action. Our analysis provides quantitative support for the qualitative assertion by Trinh et al. [3] that therapeutic doses of PSE do not affect performance. However, we also question their view that supratherapeutic doses of PSE induce an ergogenic effect.

## Methods

This review complies with the Preferred Reporting Items for Systematic Reviews and Meta-Analyses (PRISMA) statement [16]. An extensive literature search was carried out prior to statistical analyses using a random-effects model.

### Search and selection strategy

**Fig. 1:**
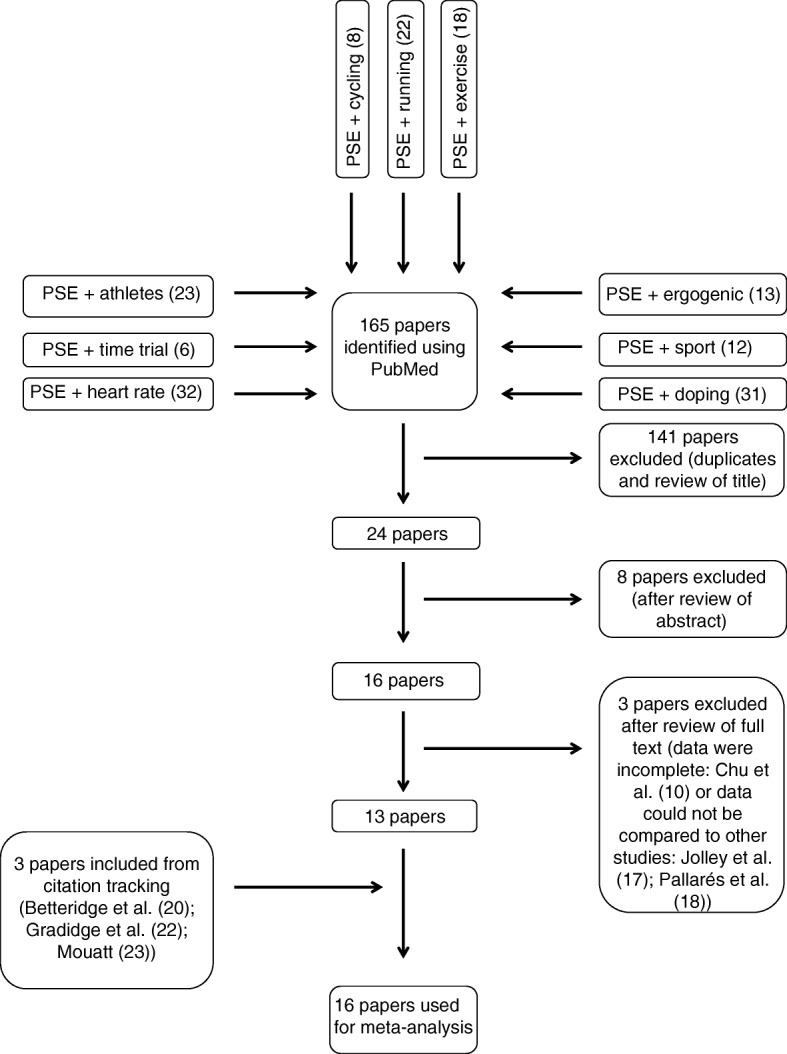
Flowchart showing the search process

Figure 1 outlines the search process. A search of the PubMed database took place in October and November 2015. After the identification of 13 eligible studies, a citation track was conducted and three more articles were found. This was combined with a search of articles’ references lists, focusing on those which had cited the original 13 articles identified. This was further supplemented by Google Scholar searches in November 2015 and August 2017. As of February 2018, no new relevant references were found using citation searches of the final 16 articles selected. Terms included in the search were ‘pseudoephedrine’ in combination with ‘heart rate’, ‘time trial’, ‘athletes’, ‘cycling’, ‘running’, ‘exercise’, ‘ergogenic’, ‘sport’, and ‘doping’. These terms were searched for in the title and the abstract of studies.

### Study selection criteria

Only articles investigating the effect of PSE on exercise performance were selected. Authors must have provided enough information to derive means and standard deviations for performance tests. Studies must have used PSE as the only substance in the intervention, and they were excluded if the substance was not specifically being investigated for its ergogenic effect [17]. Also excluded were studies investigating the effects of PSE on strength and neuromuscular coordination because data were incomparable with included articles [10, 18]. Studies that investigated other substances were included if participants were not administered both substances simultaneously [7, 12, 19]. All studies were randomised placebo-controlled trials and were conducted in a double blind crossover fashion. All participants (males and females) were deemed to be healthy. Each protocol of the 16 studies included in this meta-analysis required the participants to abstain from use of stimulants before the trials and some studies had a pre-planned meal.

### Data extraction

To estimate the effect of PSE on performance, data for the following variables were extracted from the gathered articles where available: HR, GLU, LAC, time trial (TT) performance, and RPE. Table 1 shows the characteristics of the 16 studies included in the meta-analysis. Eleven of these measured HR [7, 8, 11, 13–15, 19–23], 5 measured GLU [6, 7, 14, 15, 23], 6 measured LAC [7, 13–15, 19, 24], 9 measured TT [9, 14, 15, 19, 21–25], and 5 measured RPE [8, 12, 15, 22, 23]. Where data were presented only in figures [7, 24], authors were directly contacted for the raw data. If these were not available, the figures were enlarged and values were calculated using a ruler [26].

**Table 1:** Characteristics of studies included in the meta-analysis

Authors	Title of study	PSE dose (mg)	Time of ingestion pre exercise (min)	Washout period (days)	VO_2_ max (ml/kg/min)	No. of participants	Sex (mean age ± SD)	Type of exercise	Dominant energy source for exercise	Parameters measured (included in meta-analysis)	Subgroup for analysis	Conclusion of study
Bright et al. [6]	“Selected cardiac and metabolic responses to pseudoephedrine with exercise”	60 or 120	60	7	/	6	Male (25.5)	Approx. 12 min multistage treadmill running exercise until 85% max. HR was reached	Aerobic	blood glucose	LD, SE, YA, LW, SI, RU	No significant changes in cardiovascular or metabolic parameters.
Clemons and Crosby [8]	“Cardiopulmonary and subjective effects of a 60 mg dose of pseudoephedrine on graded treadmill exercise”	60	70	7	58.46	10	Female (20.4 ± 1.71)	Seven 3 min continuous running exercise stages with speed increasing at 19.22 m/min in each stage	Aerobic	HR, RPE	LD, SE, YA, VL, LW, SI, RU	No effect although it may augment submaximal exercise HR and slow HR recovery.
Gillies et al. [9]	“Pseudoephedrine is without ergogenic effect during prolonged exercise”	120	120	7	/	10	Male (23.3 ± 2.84)	Approx. 60 min high-intensity exercise (40 km cycling time trial)	Aerobic	TT	LD, LE, YA, LW, LI, CY	No ergogenic effect during prolonged exercise.
Swain et al. [12]	“Do pseudoephedrine or phenylpropanolamine improve maximum oxygen uptake and time to exhaustion?”	1 mg/kg or 2 mg/kg (78.62 mg or 157.24 mg)	60	7	59.52	20	Male (27.1 ± 3.73)	10 s to achieve 80 rpm in a cycling trial with test ending when subjects are unable to maintain speed after 10s	Aerobic	RPE	LD, SE, YA, VL, LW, SI, CY	No ergogenic effect.
Gill et al. [13]	“Muscular and cardiorespiratory effects of pseudoephedrine in human athletes”	180	45	7	/	22	Male (21.0 ± 2.8)	Maximal (30 s “all-out”) cycle sprint (cycling)	Anaerobic	HR, blood lactate	HD, SE, YA, LW, SI, CY	Improved peak power during maximal cycle performance.
Chester et al. [7]	“Physiological, subjective and performance effects of pseudoephedrine and phenylpropanolamine during endurance running exercise”	60 (6 doses over 36 h)	240	7	65.46	8	Male (29.58 ± 8.42)	20 min running followed by a 5000-m time trial	Aerobic	HR, blood lactate, blood glucose	HD, LE, OA, VH, LW, LI, RU	No ergogenic effect with regard to endurance running.
Hodges, et al. [11]	“Effects of pseudoephedrine on maximal cycling power and submaximal cycling efficiency”	60	90	3	56.8	11	Male (29.0 ± 8.6)	10 min cycling test (at 40% and 60% of peak power) and 30 s maximal cycle test	Aerobic and Anaerobic	HR	LD, SE, OA, VL, SW, LI, CY	No effect on anaerobic cycling performance or aerobic cycling efficiency.
Hodges, et al. [14]	“Pseudoephedrine enhances performance in 1500-m runners”	2.5 mg/kg (170 mg)	90	7	68.7	7 (1 dropout)	Male (20.1 ± 1.2)	1500-m running exercise	Aerobic	HR, blood lactate, blood glucose, TT	LD, SE, YA, VH, LW, LI, RU	Improvement (by 2.1%) in 1500-m running performance
Mouatt [23]	“The physiological effects of pseudoephedrine on endurance cycling”	2.5 mg/kg (184 mg)	90	6	66.1	10	Male (29.7 ± 7)	120 min cycling exercise at fixed intensity and 30 min self-paced time trial	Aerobic	HR, blood glucose, TT, RPE	HD, LE, OA, VH, SW, LI, CY	Increased heart rate but unchanged cycling performance during endurance cycling.
Betteridge et al. [20]	“The effect of pseudoephedrine on self-paced endurance cycling performance”	2.5 mg/kg (187.5 mg)	90	6	69	8	Male (29.0 ± 6)	150 min cycling exercise at 70% VO_2_ max	Aerobic	HR, TT	HD, LE, OA, VH, SW, LI, CY	No effect on self-paced endurance exercise performance but may affect the cardiac response to exercise.
Pritchard-Peschek et al. [15]	“Pseudoephedrine ingestion and cycling time-trial performance”	180	60	3.5	56.8	6	Male (33 ± 2)	Approx. 30 min cycling exercise at 7 kJ/kg BM workload	Aerobic	HR, blood lactate, blood glucose, TT, RPE	HD, LE, OA, VL, SW, SI, CY	Significantly improved cycling TT performance by 5.1% compared to placebo.
Berry and Wagner [21]	“Effects of pseudoephedrine on 800-m run times of female collegiate track athletes”	2.5 mg/kg (144 mg)	90	7	/	13 (2 dropouts)	Female (19.6 ± 1.3)	800-m running exercise	Aerobic	HR, TT	LD, SE, YA, LW, LI, RU	No effect on 800-m running performance.
Gradidge et al. [22]	“Effect of a therapeutic dose of pseudoephedrine on swimmers’ performance”	90 mg/day	Performance was measured after a 4-day period of ingestion of PSE	4	/	7	Male and Female (44 ± 7)	50-m sprint and 2000-m swimming exercise	Anaerobic and Aerobic	HR, TT, RPE	LD, LE, OA, SW	No major effect with regard to swimming.
Pritchard-Peschek et al. [24]	“Pseudoephedrine and preexercise feeding: influence on performance”	2.8 mg/kg (204 mg)	110	7	64.8	10	Male (30.6 ± 6.6)	Approx. 30 min cycling time trial at 7 kJ/kg BM workload	Aerobic	blood lactate, TT	HD, LE, OA, VL, LW, LI, CY	No effect on cycling TT performance of approx. 30 min.
Pritchard-Peschek et al. [25]	“The dose-response relationship between pseudoephedrine ingestion and exercise performance”	2.3 mg/kg or 2.8 mg/kg) (172.7 mg or 210.28 mg)	85	7	65	10	Male (26.5 ± 6.2)	Approx. 30 min cycling time trial at 7 kJ/kg BM workload	Aerobic	TT	HD, SE, YA, VH, LW, LI, CY	No effect on cycling TT performance.
Spence et al. [19]	“A comparison of caffeine versus pseudoephedrine on cycling time-trial performance”	180	60	2	58.9	10	Male (30 ± 2)	Approx. 60 min exercise (40-km cycling time trial)	Aerobic	HR, blood lactate, TT	HD, LE, OA, VL, SW, SI, CY	No significant improvement on cycling TT.

*PSE*, pseudoephedrine; *HR*, heart rate; *RPE*, rate of perceived exertion; *TT*, time trial; *s*, second(s); *min*, minute(s); *h*, hour; *m*, metre(s); *km*, kilometre(s); *rpm*, rotations per minute; *VO*_*2 max*_*,* maximum oxygen uptake; *BM*, body mass. Grey shading denotes study not included in the systematic review by Trinh et al. [3]. Subgroup code (see Table 3 for quantitative details): high dose/low dose (*HD/LD*); long/short exercise duration (*LE/SE*); older/younger (*OA/YA*); VO_2 max_ higher/lower (*VH/VL*); long washout/short washout (*LW/SW*); long/short pre-exercise ingestion time (*LI/SI*); cycling/running (*CY/RU*). PSE dose given as ‘mg/kg’ was converted to ‘mg’ using mean body mass of participants

### Statistical analysis

The meta-analysis was carried out using the RevMan [27] software. The inverse-variance random-effects model for meta-analyses was used as it proportionately weights studies based on the magnitude of their standard errors [28] and accounts for heterogeneity across trials [29]. Effect sizes are presented as the standardised mean difference with 95% confidence intervals and were evaluated with the scale of Hopkins et al. [30]: < 0.2 = trivial; 0.2–0.59 = small, 0.6–1.19 = moderate, 1.2–1.99 = large, 2.0–3.99 = very large, ≥ 4.0 = extremely large. To account for the crossover design of the included studies, data for experimental and placebo conditions were analysed in the manner of a parallel group trial [28]. This method can result in wider than normal confidence intervals and underweighting of studies [28] though the short half-life of PSE (≤ 8 h [31]), coupled with more extensive washout periods, reduces the confounding impact of these factors. Study heterogeneity is represented by the *I*^2^ statistic which is the variation of effects that could be attributed to differences across studies rather than to chance. Low, moderate, and high heterogeneity correspond to *I*^2^ values of 25%, 50%, and 75% respectively [32].

## Results

The 16 analysed studies included 168 participants. Most (81%) had only male participants while two (12.5%) had only female participants. One (6.5%) study included both sexes. Mean ages ranged from 18 to 38 years, with only one study [22] allowing participants up to 60 years. Participants were either competitive athletes or volunteers with an interest in sport.

### Primary effects

**Fig. 2:**
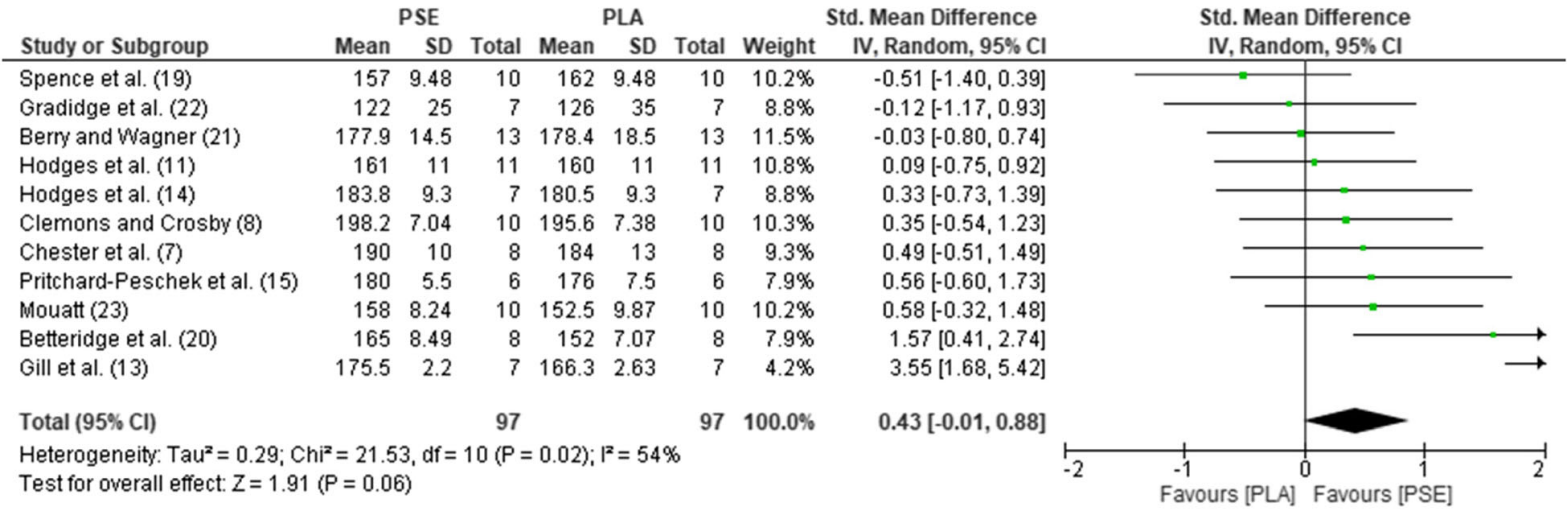
Forest plot for effects of PSE on HR with 95% confidence intervals, HR, heart rate; PSE, pseudoephedrine; PLA, placebo; SD, standard deviation; Std., standardised; IV, instrumental variables; CI, confidence interval. Positive effect sizes represent an increase in HR due to PSE.

**Fig. 3:**
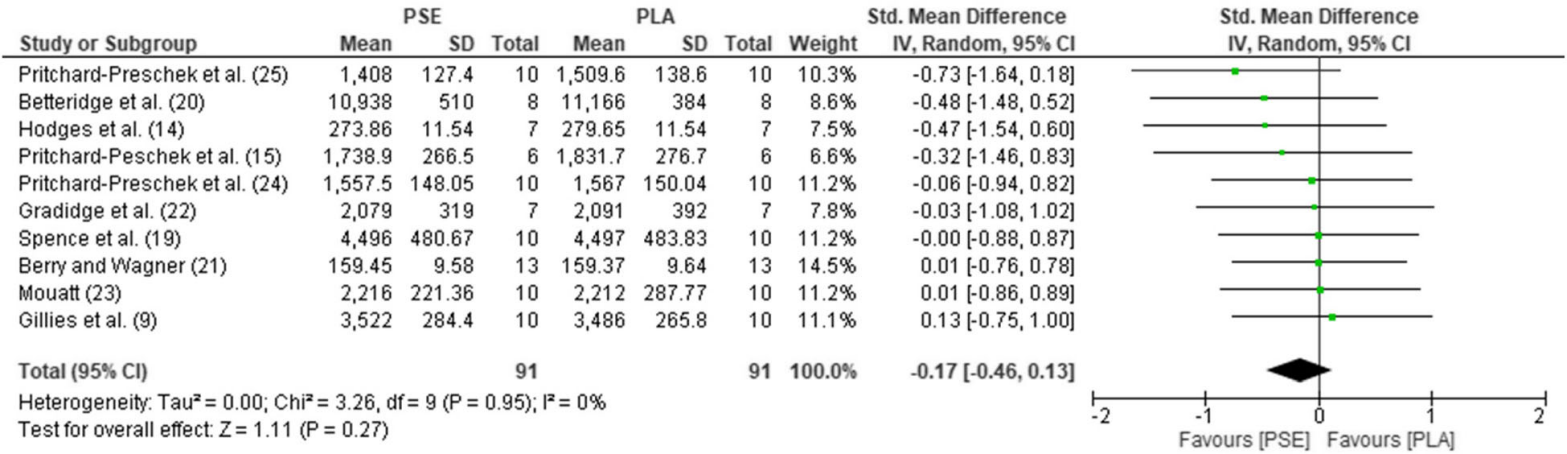
Forest plot for effects of PSE on TT with 95% confidence intervals, PSE, pseudoephedrine; PLA, placebo; SD, standard deviation; Std., standardised; IV, instrumental variables; CI, confidence interval. Negative effect sizes represent a shorter TT performance due to PSE.

**Fig. 4:**
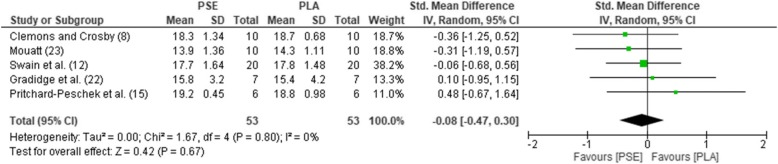
Forest plot for effects of PSE on RPE with 95% confidence intervals. RPE, rating of perceived exertion; PSE, pseudoephedrine; PLA, placebo; SD, standard deviation; Std., standardised; IV, instrumental variables; CI, confidence interval. Positive effect sizes represent an increased RPE due to PSE.

**Fig. 5:**
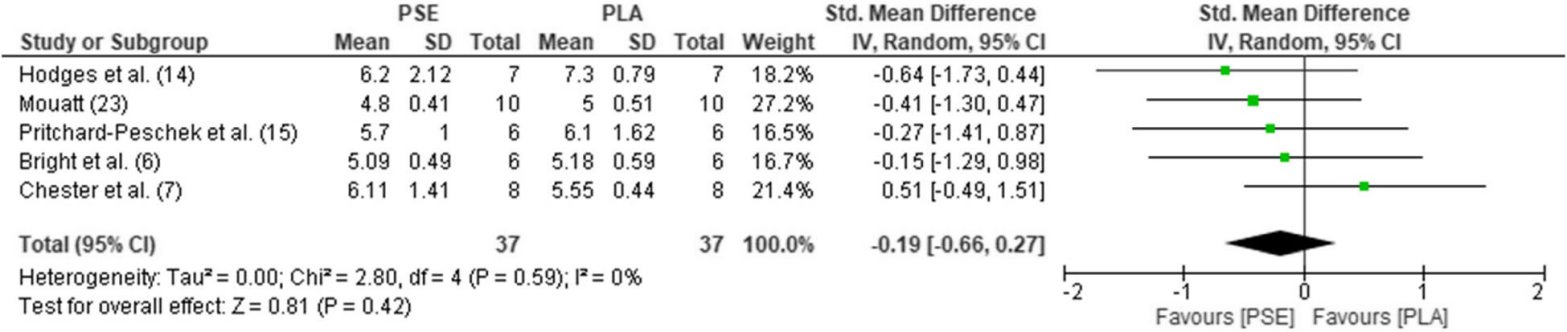
Forest plot for effects of PSE on GLU with 95% confidence intervals. GLU, blood glucose; PSE, pseudoephedrine; PLA, placebo; SD, standard deviation; Std., standardised; IV, instrumental variables; CI, confidence interval. Positive effect sizes represent an increase in GLU due to PSE

**Fig. 6:**
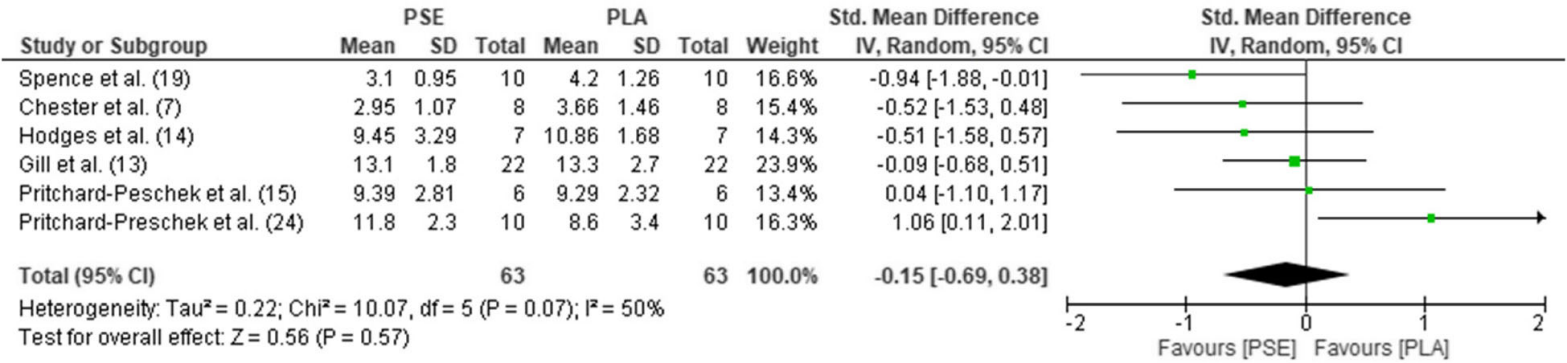
Forest plot for effects of PSE on LAC with 95% confidence intervals.LAC: Blood lactate; PSE, pseudoephedrine; PLA, placebo; SD, standard deviation; Std., standardised; IV, instrumental variables; CI, confidence interval. Positive effect sizes represent an increase in LAC due to PSE

The pooled mean estimates for the effect of PSE on HR, TT, RPE, GLU, and LAC are shown in Table 2 and Figs. 2, 3, 4, 5, and 6. Effects were generally positively or negatively trivial across parameters with the exception of HR which showed a small positive effect in favour of PSE ingestion.

**Table 2:** Effect sizes and descriptors for parameters studied

Parameter	HR	TT	RPE	GLU	LAC
Effect size (95% confidence interval)	0.43 (− 0.01, 0.88)	− 0.17 (− 0.46, 0.13)	−0.08 (− 0.47, 0.30)	−0.19 (− 0.66, 0.27)	−0.15 (− 0.69, 0.38)
Effect size descriptor	Small increase	Trivial decrease	Trivial decrease	Trivial decrease	Trivial decrease

*HR*, heart rate; *TT*, time trial; *RPE*, rate of perceived exertion; *GLU*, blood glucose; *LAC,* blood lactate

### Effects in subgroups

Subgroups, chosen by a median split, revealed important trends (Table 3). Effect sizes on HR and TT tended to be larger in well-trained (≥ 65 ml/kg/min) and younger (< 28 years) participants. Effects were also larger for shorter (< 25 min) bouts of exercise and when PSE was administered less than 90 min prior to performance. For longer duration exercise (≥ 90 mins), small effects were also apparent for RPE. There was evidence of a dose-response for TT and HR with larger doses (> 170 mg) resulting in small and moderate effect sizes respectively for these variables.

**Table 3:** Subgroup analysis

		HR			TT			RPE			GLU			LAC		
Subgroup	Median split	Effect size	Groups	N	Effect size	Groups	N	Effect size	Groups	N	Effect size	Groups	N	Effect size	Groups	N
PSE dose	> 170 mg	0.85	6	49	− 0.24	6	54	0.00	2	16	− 0.08	3	24	− 0.09	5	56
	≤ 170 mg	0.11	5	48	− 0.06	4	37	− 0.11	3	37	− 0.41	2	13	− 0.51	1	7
Exercise duration	≥ 25 mins	0.38	6	49	− 0.08	7	61	0.02	3	23	− 0.08	3	24	− 0.10	4	34
	< 25 mins	0.55	5	48	− 0.34	3	30	− 0.16	2	30	− 0.41	2	13	− 0.18	2	29
Age	> 28 years	0.32	7	60	− 0.12	6	51	0.02	3	23	− 0.08	3	24	− 0.10	4	34
	< 28 years	0.77	4	37	− 0.22	4	40	− 0.16	2	30	− 0.41	2	13	− 0.18	2	29
VO_2_ max	≥ 65 ml/kg/min	0.69	4	33	− 0.40	4	35	− 0.31	1	10	− 0.18	3	25	− 0.52	2	15
	< 65 ml/kg/min	0.07	4	37	− 0.10	3	26	− 0.06	3	36	− 0.27	1	6	0.05	3	26
Washout period (days)	≥ 7 days	0.65	5	45	− 0.19	5	50	− 0.16	2	30	− 0.07	3	21	0.00	4	47
	< 7 days	0.31	6	52	− 0.14	5	41	0.02	3	23	− 0.36	2	16	− 0.51	2	16
Pre-exercise ingestion time	≥ 90 min	0.41	6	57	− 0.10	6	58	− 0.31	1	10	− 0.18	3	25	0.03	3	25
	< 90 min	0.54	5	40	− 0.34	3	26	− 0.06	3	36	− 0.21	2	12	− 0.30	3	38
Mode of exercise	Cycling	0.77	6	52	− 0.09	6	54	− 0.04	3	36	− 0.36	2	16	0.00	4	48
	Running	0.18	5	45	− 0.16	2	20	− 0.36	1	10	− 0.07	3	21	− 0.52	2	15

*PSE*, pseudoephedrine; *HR*, heart rate; *RPE*, rate of perceived exertion; *TT*, time trial; *GLU*, blood glucose; *LAC*, blood lactate; min, minute(s); VO_2max_, maximum oxygen uptake

## Discussion

Our results quantitatively demonstrate that PSE causes a small increase in HR during exercise. In terms of the other parameters studied, there were trivial improvements in time trial performance, a trivial reduction in RPE and trivial decreases in GLU and LAC levels during exercise. It could be argued that these equivocal findings suggest a meta-analysis would better wait until a larger number of studies have been performed, thus leading to a more robust conclusion. However, the intriguing subgroup analyses argue against this. Effect sizes tended to be larger in just those athletes of most concern to anti-doping agencies (younger and well-trained athletes). They also suggest an optimal time and activity to take the drug, indicating PSE is most effectively administered less than 90 min before a short bout of exercise of less than 25 min. Of particular concern is that our subgroup analysis confirms the qualitative review [3] that larger doses (> 170 mg) are likely to be the most effective in improving performance. However, this was accompanied by a larger effect on increasing HR. A recent study looking at neuromuscular performance effects using these more effective higher PSE doses (180 mg) noted adverse side effects such as tachycardia and heart palpitations 24 h after exercise [18]. This suggests it will be increasingly difficult to get ethical approval to test the most effective doses of PSE, making it important to carry out the most complete analysis of the studies that have already been performed.

### Comparison to previous systematic reviews

The initial search for our systematic review was carried out at approximately the same time as that of the recent systematic review by Trinh et al. [3] and subsequent searches uncovered no additional studies of interest. However, the studies deemed appropriate for detailed analysis were different. Whilst our search confirmed and agreed with the many of the studies chosen by Trinh et al. [3], we included some additional publications. As our enhanced sample enabled the meta-analysis that Trinh et al., [3] felt not to be justified, we feel it is important to justify the rationale for the additional papers chosen.

Both reviews focused on the performance effects of PSE using a randomised-controlled trial approach. As our study was designed to enable a meta-analysis, it was restricted to sports performances that had a time trial component and/or included quantitative measures that could inform potential underlying mechanisms (such as LAC, GLU, HR, and RPE). This biased our search to include only those studies that focused on sports events with an aerobic component. This approach ruled out one article included by Trinh et al. [3], a study by Chu et al. [10] showing that a moderate dose (120 mg) of PSE did not alter muscle action strength or anaerobic power. It also ruled out a recent study published after both systematic searches. In 2015, Pallarés et al. [18] measured bench press and full squat exercise performance against four incremental loads (25%, 50%, 75%, and 90% one repetition maximum). No effects were seen except in the highest dose studied (180 mg) where PSE seemingly increased lower body muscle contraction velocity.

Trinh et al. [3] conducted their analysis on only 10 studies and concluded that the data were insufficient and too variable to enable a meta-analysis. We feel that, at least in part, this conclusion is based on the combination of a flawed search strategy and an overly restrictive view of which articles to select from that search. We found seven additional papers omitted by Trinh et al. [3], all of which used randomised placebo designs to assess the effect of PSE on aspects of performance. On this basis, these studies could, in principle, fit the criteria used by Trinh et al. [3]. These articles are highlighted in Table 1 and owing to their importance to our final meta-analysis, it is crucial that we justify their inclusion individually (see discussion in Table 4).

**Table 4:** Characteristics of studies included in this meta-analysis that were not included in Trinh et al. [3] systematic review

Authors	Title of study	Rationale for inclusion in the meta-analysis
Bright et al. [6]	“Selected cardiac and metabolic responses to pseudoephedrine with exercise”	This paper studied the cardiac and metabolic responses to pseudoephedrine with exercise. As GLU after exercise was measured (a non-significant small decrease was seen), it was included in our study. The relevant performance measure was ‘time to reach 85% of maximum HR’ during submaximal exercise, with no effect being seen. The inclusion criteria of Trinh et al. accounted for *“*any enhancement in sport above baseline such as timing, strength, time to fatigue and/or respiratory enhancement*”.* Although it did not fit our time trial criteria, it is at least arguable that time to reach 85% of maximum HR fits those of Trinh et al.
Clemons and Crosby [8]	“Cardiopulmonary and subjective effects of a 60 mg dose of pseudoephedrine on graded treadmill exercise”	This evaluated the cardiopulmonary and subjective effects of a 60 mg dose of PSE on graded treadmill running. The RPE and HR data recorded during exercise were included in our meta-analysis. However, the time to exhaustion was not, as it was a graded exercise test rather than a time trial. Despite this, it would seem that time to fatigue on a treadmill would fit well with the Trinh et al. criteria, so it was not clear why they did not discuss this research.
Mouatt [23]	“The physiological effects of pseudoephedrine on endurance cycling”	This study looked at the effects of high dose (2.5 mg/kg ≅ 184 mg total) PSE on endurance cycling. This comprehensive randomised controlled trial was included in our analysis as it measured HR, GLU, RPE and TT duration. A difficult study to find owing to it only being published as an MSc. thesis, it is freely and readily available in open access form via standard search engines (Google etc.). Nevertheless, as a research thesis from a well-recognised university (Massey), supervised by a well-published author in the sports and exercise science field (Toby Mündel), we feel it is appropriate to add to our analysis.
Betteridge et al. [20]	“The effect of pseudoephedrine on self-paced endurance cycling performance”	This used a randomised controlled study design to measure HR, GLU, LAC, RPE, and TT duration after a high dose PSE. Heart rate and TT were included in our meta-analysis; however, changes in GLU and LAC values could not be used as they were not reported in sufficient detail. The European Journal of Sports Science is the official journal of the European College of Sports Science, but was not listed in Medline until 2013 so the search strategy of Trinh et al. would not have uncovered it as their search strategy excluded sports and exercise science databases. Trinh et al.’s criteria also did not include citation or reference searches of the final selected papers, which might have rectified this omission as the relevant article was commented on in the discussion of one of the studies [25] that was cited by Trinh et al.
Gradidge et al. [22]	“Effect of a therapeutic dose of pseudoephedrine on swimmers’ performance”	This paper explored the effect of a low dose of PSE on swim performance (TT, RPE, and HR data were included in our analysis). This double blind randomised controlled trial was published in the South African Journal of Sports Medicine, which is absent from the search database used by Trinh et al. [3].
Pritchard-Peschek [24]	“Pseudoephedrine and preexercise feeding: influence on performance”	An apparent inconsistency occurred in the selection of studies from Pritchard-Peschek and collaborators between ourselves and Trinh et al. [3]. Between 2010 and 2014, this group published three randomised controlled trials on PSE and exercise performance. All included TT data and so were included in our meta-analysis. However, Trinh et al., only included the papers published in 2010 [15] and 2014 [25], despite the 2013 paper being published in Medicine and Science in Sports and Exercise and hence readily accessible by Medline. Their 2013 paper [24] had a similar protocol to those in 2010 and 2014 [25], but with the addition of a pre-ingestion meal group. However, this additional group could be easily removed for consistency with the other two studies and so was included in our analysis.
Spence et al. [19]	“A comparison of caffeine versus pseudoephedrine on cycling time-trial performance”	This paper was analysed by Trinh et al., but specifically excluded from their analysis. It compared caffeine and PSE in cycling time trial performance. Trinh et al. excluded the study as they stated it “focuses on differences between effects of caffeine and PSE”. However, in their inclusion criteria, they note that “studies that looked at other substances were included if athletes were not administered both substances simultaneously”. This article outlined three experimental arms (caffeine, PSE, and placebo) in a cross-over study with adequate wash out periods between trials. It is true that the authors focus on the differences between caffeine and PSE in their discussion, but they give full statistics (means, standard deviations, and effect sizes) for a comparison between PSE and placebo. In light of this, by their own criteria, we feel that Trinh et al. should not have excluded this study.

Given that Trinh et al. [3] only included 10 articles in their final qualitative synthesis, the exclusion of seven relevant studies represents a significant fraction of the available literature. *Crucially, all seven studies excluded showed no effect of PSE on performance*. This may not be related to a dose effect as three studies [6, 8, 22] were at low (clinically approved) doses and four were at supratherapeutic doses [19, 20, 23, 24]. It is possible that excluding such a large fraction of data biased the final conclusion of that review, particularly the comment that “qualitative analysis showed overall positive results in favour of PSE over placebo for PSE doses ≥180 mg or 2.5 mg/kg”. Of the 10 studies included by Trinh et al. [3], all three high dose studies showed an ergogenic effect, and all seven lower dose studies showed PSE to be ineffective, making their conclusion reasonable. However, adding the seven omitted studies would significantly weaken this argument as only 3 out of 7 high dose studies demonstrate a positive effect of PSE. Therefore, although there is clearly an increase in HR during exercise due to PSE, we are more equivocal than Trinh et al. [3] about the drug’s positive ergogenic effects, even at high doses. Including these new articles does, however, strongly favour the conclusion that when taken at clinically recommended doses, PSE has only a very minor effect on HR and no ergogenic effect in terms of performance.

### Relevance for putative mechanism of any performance benefit

Our analysis suggests that only at high doses does PSE have the potential to enhance sports performance. It also sheds some light on to the possible mechanism that could be operating. A previous meta-analysis demonstrated that, at rest, PSE caused a statistically significant small increase in systolic blood pressure (1 mmHg) and HR (3 beats/min), although diastolic blood pressure did not change. We found 11 studies reporting HR changes following PSE ingestion during exercise. Our data showed that this mean HR increase is maintained during exercise with the largest increase being 13 beats/min [20]. The subgroup analyses showed that the biggest effect sizes were seen at high doses and in athletes with high maximal oxygen uptake (VO_2_ max). Three individual studies showed a performance effect [13–15]. Gill et al. [13] showed that HR increased significantly from 166 to 175 beats/min, and Hodges et al. [14] demonstrated a non-significant increase from 185 to 190 beats/min. Similarly, Pritchard-Peschek et al. [15] also reported a non-significant increase was from 176 to 180 beats/min. Given that studies that showed no performance increase showed at least as large and as significant HR increases during exercise, it seems unlikely that—in and of itself—changes in HR underpin any performance enhancement.

In relation to RPE, GLU, and LAC levels in exercise, a lack of data is more challenging to overcome with only between five and seven studies reporting sufficient information. We observed a small, trivial decrease in all of these parameters. However, it is worth exploring the individual studies, as the statistical power of pairing individuals in a crossover study is lost during a meta-analysis.

For RPE, none of the five studies included showed meaningful differences between PSE and placebo [8, 12, 15, 22, 23]. An additional study did not report values, but did state that there were no significant differences [7]. Glucose levels were not significantly different in four of the five studies included in the meta-analysis [6, 7, 14, 23]. An additional study, not part of the analysis as it reported no values, again stated a lack of any PSE effect on GLU [25]. However, one study did report increased GLU levels post exercise following PSE treatment [15]. This was one of the few studies that also showed a performance effect (decreased time in cycling TT). Interestingly in this case, it was the pre-exercise GLU level that correlated with the increased performance in the cycling time trial. Lactate levels did not significantly change in five of the seven studies included in the meta-analysis [7, 13–15, 23]. However, they did significantly decrease in two studies [19, 24]. In one study, LAC levels were not reported, the authors nonetheless stating there was no significant change [25].

Given that only 3 of the 16 studies included in our analysis showed a performance benefit, it is worth exploring in detail, which secondary parameters changed in these studies to see if this can inform mechanism. Gill et al. [13] measured an increase in maximum torque in an isometric knee extension and an improvement in peak power during maximal cycle performance in 22 healthy male volunteers. In terms of lung function, small, but significant, increases were seen in forced vital capacity (FVC) and forced expired volume in 1 s (FEV) following ingestion of PSE. These are consistent with the well-characterised role of PSE in stimulating the sympathetic nervous system and acting as a bronchodilator [33]. This is unlikely to explain the effect on peak power observed here, nor is a small increase in FVC and FEV likely to improve sports performance in endurance events given the lack of consistent ergogenic effect of drugs that are far more effective in increasing lung function such as salbutamol [34].

Hodges et al. [14] found that PSE significantly decreased time to completion of a 1500 m time trial in 7 healthy male subjects. However, no other measured parameters (HR, LAC, GLU, arterial O_2_ partial pressure, arterial carbon dioxide partial pressure and arterial oxygen saturation) were significantly altered.

Pritchard-Peschek et al. [15] reported a significant improvement in a cycling TT performance following PSE in six trained male cyclists and triathletes. As previously noted, this study reported increased post exercise GLU levels following PSE treatment. No significant PSE effect was found on LAC, blood pH, substrate oxidation, RPE, or HR. PSE did significantly increase plasma norepinephrine concentrations, an expected outcome for a drug that has indirect agonist activity on cardiac β receptors and peripheral α_1_ receptors, through release of norepinephrine from the cytoplasmic pool [14]. However, the validity of any ergogenic effects of this increased β receptor activity is undermined by the two subsequent similar studies from this group, which used larger sample sizes (*n* = 10) and showed no performance benefit despite an increase in plasma norepinephrine [24, 25].

Readily available drugs used as decongestants that are not banned by WADA such as phenylephrine act directly on peripheral α receptors and have limited ability to cross the blood-brain barrier and/or act as a central stimulant [2]. PSE is more lipid soluble and is therefore more accessible to the central nervous system. Consequently, it can, in principle, act as both a peripheral or central stimulant. However, the biochemical, physiological, and psychological data in our systematic review and meta-analysis fail to give a consistent explanation to underpin a possible ergogenic mechanism. Heart rate did increase, however, in most studies there was no accompanying performance benefit; indeed in some studies which showed a performance benefit, there is no significant heart rate change. A few studies show plasma metabolite changes (GLU/LAC) that might suggest improved substrate or oxygen utilisation. However, other studies show no metabolite changes even when there is a performance benefit. Unlike other WADA banned stimulants such as amphetamines [35], perception of effort (RPE) is completely unchanged by PSE at low or high doses, irrespective of any performance benefit.

### Rationale for WADA listing pseudoephedrine as a prohibited doping substance

The WADA Prohibited List may include any substance that satisfies any two of the following three criteria: (i) it has the potential to enhance or enhances sport performance; (ii) it represents an actual or potential health risk to the athlete; (iii) it violates the spirit of sport. Apparently, PSE fulfilled these criteria and was banned until 2004, did not fulfil them between 2004 and 2010 (when it was removed from the banned list), and then fulfilled them again after 2010 (when it returned to the banned list). Currently, PSE is only banned in competition. A doping offence is committed if an athlete has a urine PSE concentration of greater than 150 μg/ml. Even given the biological variability of single point measurements, this level is high enough that it should not be possible to produce a positive urine test if an athlete discontinues a therapeutic dose of PSE more than 24 h before competition. However, it is possible, though not guaranteed, to exceed these levels within 24 h of taking PSE at the normal therapeutic dose [9], and it is impossible not to exceed them when on a supratherapeutic dose [24].

WADA monitored PSE use in doping samples when it was not banned from 2004 to 2009. WADA’s case for reintroducing the PSE ban in 2010 was made in a Q and A statement published as part of the 2010 prohibited list [36]:

“Results of the Monitoring Program over the past five years have shown a sustained increase in samples containing pseudoephedrine. The program indicated clear abuse of this substance with high concentrations in a number of sports and regions. In addition, available literature shows scientific evidence of the performance-enhancing effects of pseudoephedrine beyond certain doses.”

Some increase in PSE use would be expected when the ban was lifted given that the best drug to treat nasal decongestion in competition was now freely available to athletes without the threat of sanction. Presumably, the geographic and sport-specific nature of the increase argued against this more benign interpretation. An additional concern, not specifically noted by WADA, but stated by some anti-doping researchers, is that one of PSE’s minor metabolites, norpseudoephedrine (cathine), was on the banned list during this period. Athletes could therefore claim a failed cathine doping result was a consequence of taking the now permitted PSE. PSE use would, therefore, mask cathine abuse [37].

However, our systematic review does question WADA’s statement that “available literature shows scientific evidence of the performance-enhancing effects of pseudoephedrine beyond certain doses.” A research article would need to have been published between 2004 and 2009 to inform this change of policy. In this period, our search uncovered three studies reporting no performance effect [7, 14, 23] and only one coming to the contrary view [14]. That study was published in 2006 and is the only paper WADA cite in the 2004–2009 period showing a performance benefit in justification of their decision [38]. It showed a performance benefit based on only six UK college 1500 m runners, the fastest running over 4:15 min for the distance. The International Association of Athletics Federations (IAAF) qualifying standard for this event in the 2016 Olympics was 3:36 min, making this subject group far from elite. Given that PSE is not banned out of competition, a WADA-approved study in elite athletes would be beneficial to support the current policy.

Given the difficulty of taking measurements in elite athletes, it is possible that WADA treat the systematic abuse of a drug by elite athletes as partial evidence for its efficacy in that subject group. The use of a higher than necessary dose of a medicine (or even the use at all of a medicine where there is no clinical need) is also considered to be against the “spirit of sport”. A similar rationale was presumably used for the more infamous 2016 banning of the cardiac drug meldonium once the extremely widespread use of it amongst Eastern European athletes became known [39], despite the poor evidence base for its performance-enhancing effect. In the case of PSE, there is the added concern, that in some countries, to get access to the higher doses, athletes need to circumvent government regulations designed to combat the production of illegal recreational drugs [2].

## Conclusions

In contrast to a previous systematic review [3], our analysis has shown that any performance benefit of PSE is marginal at best, and certainly less than the well-characterised permitted stimulant caffeine [40]. However, a small performance benefit at high doses in elite athletes still cannot be completely ruled out at present.

## Abbreviations

ES: Effect sizes
FEV: Forced expired volume
FVC: Forced vital capacity
GLU: blood glucose concentration
HR: Heart rate
IAAF: International Association of Athletics Federations
LAC: Blood lactate concentration
PLA: Placebo
PRISMA: Preferred Reporting Items for Systematic Reviews and Meta-Analyses
PSE: Pseudoephedrine
RPE: Rate of perceived exertion
TT: Time trial
WADA: World Anti-Doping Agency
